# Effects of Intranasal and Oral *Bordetella bronchiseptica* Vaccination on the Behavioral and Olfactory Capabilities of Detection Dogs

**DOI:** 10.3389/fvets.2022.882424

**Published:** 2022-05-18

**Authors:** Amanda Collins, Rachel A. Bear, Amritha Mallikarjun, Sarah A. Kane, Jennifer L. Essler, Patricia Kaynaroglu, Rebecca Feuer, Jordan G. Smith, Cynthia M. Otto

**Affiliations:** ^1^Penn Vet Working Dog Center, School of Veterinary Medicine, University of Pennsylvania, Philadelphia, PA, United States; ^2^Department of Clinical Science and Advanced Medicine, School of Veterinary Medicine, University of Pennsylvania, Philadelphia, PA, United States

**Keywords:** *Bordetella bronchiseptica*, working dogs, detection dogs, vaccination effects, olfaction

## Abstract

The bacterium *Bordetella bronchiseptica* is responsible for serious respiratory disease in dogs, most often associated with ‘kennel cough’ (canine infectious tracheobronchitis). It is recommended that dogs are vaccinated against the bacterium every 6–12 months, either by oral or intranasal administration. Any impairment of dogs' olfactory capabilities due to medical treatments may impact their efficiency and accuracy in their jobs. This study examined (1) the effect of intranasal and oral vaccines on the olfactory capabilities of detection dogs; as well as (1) effects of the vaccines on canine behavior. Dogs that were vaccinated initially with the oral and 28 days later with intranasal *B. bronchiseptica* were generally slower to find the target odor than the dogs that were assigned intranasal then oral vaccine. This result prompted a second between-subjects study to further investigate any impact of intranasal administration of the *B. bronchiseptica* vaccine on the olfactory capabilities of dogs. The intranasal vaccine was of particular interest due to its prevalent use and potential for nasal inflammation leading to decreased olfactory capabilities. Neither odor threshold nor time spent searching for odor were affected by the intranasal vaccine. Behavioral analyses showed that behaviors associated with the dogs' positive and negative motivation affected their time spent finding the target odor; this suggests that behavior should be considered in future studies of olfactory performance.

## Introduction

Scent detection dogs are used for a broad range of purposes, including drugs and explosives detection (1), medical detection (2, 3), and search and rescue (4). These dogs often deploy with little to no prior notice. As such, it is important to determine whether regular medical treatments for these dogs have the potential to cause olfactory-related side effects, as any impacts to dogs' olfactory capabilities could hamper their ability to conduct detection work safely and effectively. While several studies have demonstrated that different drugs and vaccines can cause hyposmia or anosmia in humans (5, 6), little is known about the potential effect of drugs and vaccines on canine olfaction. It is particularly important to assess the effect of regularly administered preventatives and vaccines, as working dogs could potentially deploy shortly after their administration.

*Bordetella bronchiseptica* is a Gram-negative bacterium that causes respiratory disease in dogs, swine, and rabbits, and is a major contributor to canine infectious tracheobronchitis (colloquially known as “kennel cough”) (7). While *B. bronchiseptica* is rarely the causative agent of disease in humans, *B. pertussis*, a related *Bordetella* bacterium, causes whooping cough in humans (8). These microorganisms target the ciliated respiratory epithelium and adhere to the cell surface, causing self-limiting ciliostasis and interference with secretory and physiological processes, all of which resolve with time (9). *B. bronchiseptica* can result in hyperplasia and metaplasia of the nasal epithelium, inflammatory cell infiltration of the submucosa, production of local secretory IgA and IgG antibodies, and epithelial degeneration and necrosis of the nasal septa, producing localized inflammation (10, 11). As such, it is crucial to prevent working dogs from contracting and spreading this disease.

One way to prevent *B. bronchiseptica* in dogs is to implement a regular vaccination schedule; however, it is unknown if the vaccine could lead to hyposmia or anosmia after inoculation. Vaccination for *B. bronchiseptica* is recommended every 6–12 months (12). Both the live avirulent intranasal and oral vaccines provide protection against infection; however, the intranasal vaccination is reportedly more effective at decreasing clinical symptoms of disease (13, 14). The intranasal vaccine directly contacts the nasal mucosal epithelium and elicits a local and rapid mucosal immune response, which may cause localized inflammation due the innate and adaptive immune responses (14). In humans, inflammation of the nasal epithelium, as seen in chronic rhinosinusitis, can result in hyposmia/anosmia (15).

Interestingly, increased cellular disorganization and goblet cell activity, infiltration of immune cells, and epithelial metaplasia are characteristics of both chronic rhinosinusitis in humans and of *B. bronchiseptica* in mammals (16). Other infectious agents that target respiratory epithelium, like canine parainfluenza and distemper viruses, can cause nasal inflammation, mucosal secretions, and/or vascular congestion that can lead to conductive anosmias in dogs (17–19). As such, it is possible that the *B. bronchiseptica* vaccine could lead to decreased odor detection ability in dogs.

Impairment of canine olfactory function could affect accuracy, precision, and/or efficiency when dogs search for target odor. Thus, the aim of this set of studies was to assess the effectiveness of the oral and intranasal *Bordetella bronchiseptica* vaccination, and the potential olfactory and behavioral impacts it may have on odor detection in trained detection dogs. The first study (Study 1: Oral and intranasal vaccine crossover study) compared intranasal to oral *Bordetella bronchiseptica* vaccine in a cross-over study carried out over 48 days. No significant difference was found overall between the oral and intranasal vaccine; however, this study raised questions as to whether the order of vaccine administration and short duration of washout may have spuriously influenced the olfactory performance.

As such, the second study (Study 2: intranasal vaccine between-subjects study) built on the initial findings of Study 1 and increased the clinical relevance by comparing intranasal *Bordetella bronchiseptica* vaccine to placebo and a contemporaneous control (no treatment) in a between-subjects design over a period of 29 days post-vaccination.

## Subjects

We utilized dogs trained by Penn Vet Working Dog Center (PVWDC) trainers, some owned by the University of Pennsylvania and some personally owned by PVWDC employees. Prior to testing all dogs were trained to detect Universal Detector Calibrant (UDC), a synthetic training odor which allows dogs to learn odor detection skills without compromising any future target odor they may require for their career (20–22). Dogs were also trained on the mechanics of the individual scent wheels that were utilized during baseline assessments and testing. All dogs were trained by PVWDC staff either through their enrollment in the PVWDC olfactory detection training program or through the citizen science program, which trains dogs and their owners the basics of scent detection using UDC.

### Study 1: Crossover Oral and Intranasal Vaccine Study

This study was approved by the University of Pennsylvania Institutional Animal Care and Use Committee (Protocol # 806525).

Sixteen dogs, all owned by the PVWDC and enrolled in the olfactory detection training program were utilized for this study (see Supplementary Table 1 for breeds and ages of participants). All dogs had been routinely vaccinated against *B. bronchiseptica* prior to the onset of the study.

### Study 2: Between-Subjects Intranasal Vaccine Study

This study was approved by the University of Pennsylvania Institutional Animal Care and Use Committee (Protocols #806918 for privately-owned dogs and #806894 for dogs owned by the PVWDC). Prior to the privately-owned dogs' participation in the study, the owners completed a consent form.

Twenty-four dogs were used in this study, sixteen of which dogs were working-dogs-in-training at the PVWDC, and eight were privately-owned dogs (see Supplementary Table 2 for breeds and ages of participants). None of these dogs had been vaccinated for *Bordetella bronchiseptica* for at least two months prior to the study onset.

A sample size calculation predicted that if eight dogs received the intranasal Bordetella vaccine, eight dogs received diluent, saline, intranasally and four received no treatment (control), for a total of 20 dogs, the study would have adequate power to detect a difference between groups. We included four extra dogs (for a total of 24) so the study would still have adequate power if illness, injury, failure to meet study training requirements, or other extenuating circumstances occurred that may have prevented a dog's continued participation in the study.

## Materials and Methods

### Study Design

#### Study 1: Crossover Oral and Intranasal Vaccine Study

In this block-randomized, cross-over study, the intranasal *B. bronchiseptica* vaccine was administered to half of the dogs (P1) and the oral *B. bronchiseptica* vaccine was administered to the remaining population (P2) on day 1 at time 0 (D1T0). The day prior to administration was designated as day 0 (D0). Olfactory detection acuity was tested on D0T0 (baseline), D1T4 (4 hours post), D2T0 (1 day post), D4T0 (3 days post), D8T0 (7 days post), and D11T0 (10 days post). After a 28-day washout period, the intranasal Bordetella vaccine was administered to P2 and the oral Bordetella vaccine was administered to P1. The testing protocol was repeated, and olfactory detection acuity was tested prior to and following vaccination as described above.

#### Study 2: Between-Subjects Intranasal Vaccine Study

In comparison to the previous study, this study used a between-subjects design; one group of dogs received the vaccine, one received intranasal saline, and one group received no treatment. Additionally, the dogs' performance was measured over 28 days post first vaccination rather than 10 days. Prior to vaccine administration, dogs completed 3 days of baseline testing on the wheel. In the first study at day 10, vaccinated dogs took a significantly longer time when they searched on the wheel than they did one day prior to vaccination. These additional baseline days in study 2 provide more data to account for potentially day to day variation in the unvaccinated baseline comparison for dogs' length of search post-vaccination.

Post vaccine administration, the dogs were scheduled to complete a single test trial at predetermined intervals, based on data from our previous study (Days 0, 7, 10, 14, 21, and 28).

### Training

To participate in the respective studies, each dog was required to demonstrate a trained final response when they detected UDC. This final response consisted of either a Sit alert (sitting in front of the port containing target odor), a Down alert (laying down in front of the port containing target odor) or a Stand-and-stare alert (standing with their nose within 2 inches of the port containing target odor for at least 2 s) [see (21), for more information about alert types and differences between alert types]. Dogs were trained using positive reinforcement, with a change of behavior at the UDC port marked by a clicker and a treat or toy reward. Small variations in training exist between studies due to resources available. The first study utilized a twelve-port scent wheel which was spun between trials and all dogs were run by the same handler, with the exception of one dog. The second study utilized an eight-port scent wheel in which ports were exchanged between the wheel's arms between trials and 16 of the dogs were run by the same handler. The remaining eight dogs were run by a secondary handler due to these dogs' sensitivity to novel handlers.

### Odor Stimuli

The target odor for both studies was the same in composition and concentration. The lowest concentration of UDC available was used (48 mil with a 1.6 mm hole, dissipation rate = 0.27 ng/min). The UDC provides a constant dissipation rate, ensuring that between sessions the difficulty of detection remained the same. Only one port contained the target odor in each trial, while the rest of the ports contained controls or distractors, both of which had also been included in detection training prior to the beginning of both studies. Control ports contained only the medium (powder) which is used to hold the UDC odor, and the same outer packaging the target UDC is placed in. Distractors included objects commonly found in and around the lab such as vinyl gloves, paper clips, paper towels, tape, and cotton balls. Five controls and six distractors were used in the first study while six controls and only one distractor were used in the second due to the difference in the number of ports on the scent wheels used in studies 1 and 2.

### Randomization of Participants

In Study 1, dogs were randomly assigned to two groups for order of treatment.

In Study 2, the 24 dogs were assigned to the two treatment groups and control group, balancing for breed, age, odor detection ability, and wheel mechanics (task performance ability, including checking every port and holding a 3-second alert at the target odor on the scent wheel). Odor detection ability was calculated using a d-prime score based on the dogs' training data from the past 5–7 months (depending on when the dog began training at the PVWDC). The wheel mechanics score was determined by three independent raters who ranked the dogs with which they were familiar from best to worst mechanics. These wheel mechanics scores were combined to create a single rank score for each dog (see Supplementary Table 3).

### Wheel Procedure

For both studies, dogs were run out of sight of handlers to avoid visual cues. Sessions were video recorded and were streamed to a device where the handler could see the dog searching in real time. A positive response to UDC odor was marked by the trainer with a conditioned secondary reinforcer (i.e., a clicker), then rewarded with praise and food or a toy. False negatives (passing the target odor) and false positives (alerting at the incorrect odor) were ignored. Procedure differs slightly between studies 1 and 2 due to changed standards of training at the PVWDC.

#### Study 1: Crossover Oral and Intranasal Vaccine Study

During sample placement, dogs were blocked from the trial area to prevent visual observation. During the trial, the dog searched the wheel independently, while the trainer remained behind a closed door to prevent human cues and bias. Barriers blocked off the room to decrease the number of distractors present and create a smaller search area. The dogs were free to search the wheel in any direction.

#### Study 2: Between-Subjects Intranasal Vaccine Study

**Figure 1:**
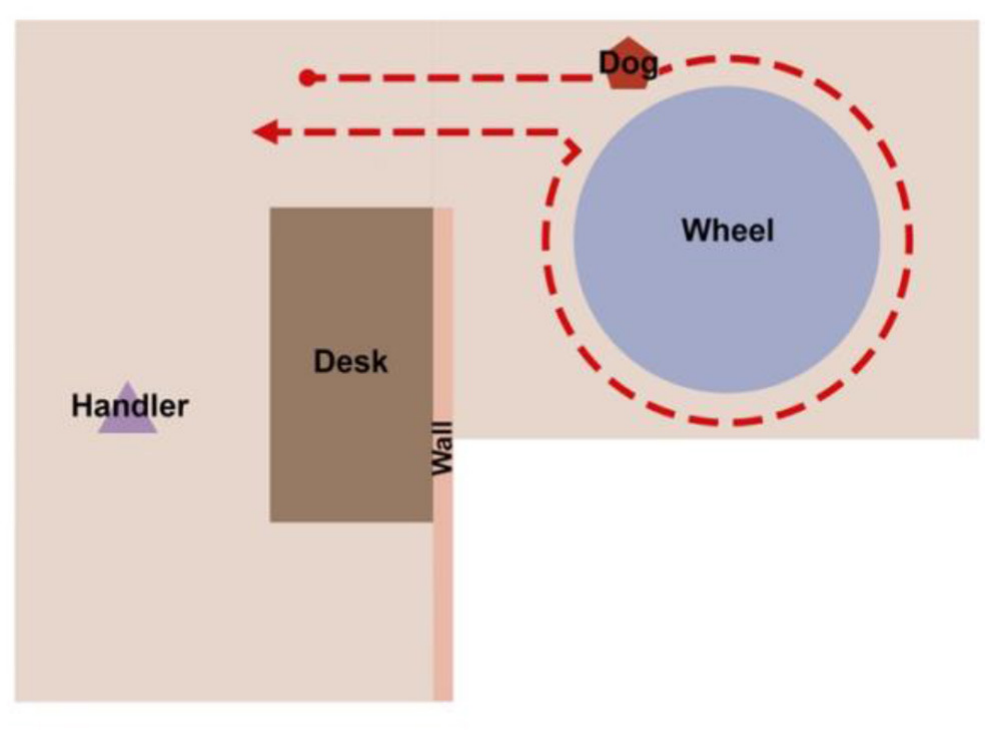
A diagram of the wheel room setup and the positions of the handler and the dog. The handler is out of sight of the dog while the dog searches on the wheel.

For each trial on the scent wheel, the dog was brought into the wheel room and their respective handler sent them to search on the wheel (see Figure 1). In this version, dogs were required to search the wheel in a specific direction, which made trial duration and statistical computations simpler. The handler was out of sight of the dog. Position of the target odor was randomized before testing sessions began and did not vary between dogs. If the dog performed their trained final response (Sit, Down, or 2-s stand and stare), the handler marked the behavior with a click and rewarded the dog. If the dog passed the target odor, an experimenter changed the odor in the target port to an increased concentration of UDC (1/16 x needle) but did not change the position of the target odor in the wheel. The handler then re-sent the dog to search on the wheel. If the dog passed the target odor again, the experimenters either moved on to the next trial, in the case of Baseline Testing, or ended the session, in the case of Post-Vaccination Testing.

### Baseline

Only one baseline session was conducted prior to vaccination in Study 1, while 3 days of baseline testing occurred in Study 2.

In the first study, baseline analysis was identical to a testing scenario, whereas in the second study the dogs were given multiple trials to find odor. Each session consisted of three trials; the position of the target odor varied between each trial, but every dog completed the same 3 trials in the same order each session. The position of the target odor was randomized and was in one of three randomly selected positions with no repeats within sessions (either port 3, 5 or 7). Each dog was brought into the wheel room and their respective handler sent them to search on the wheel. If the dog performed their trained final response (Sit, Down, or 3-s stand and stare), the handler marked the behavior with a click and rewarded the dog. If the dog passed the target odor, an experimenter changed the odor in the target port to an increased concentration of UDC (the target port remained in the same position) and the handler re-sent the dog to search on the wheel. If the dog passed the target odor again, the experimenters moved on to the next trial, where the dog was once again asked to find the lowest concentration of UDC.

### Vaccine Administration

Vaccine administration for each study varied due to route of administration and the dogs involved. The individual processes are described below.

#### Study 1: Crossover Oral and Intranasal Vaccine Study

For the oral Bordetella vaccination, the dog was lightly restrained and a 1 mL dose was administered with a syringe orally into the right buccal pouch. For the Bordetella intranasal vaccination, the vaccine was resuspended in the accompanying sterile diluent in the syringe with the attached applicator tip. The dog was lightly restrained. After a trained cue, the head was stabilized and 0.5 mL of vaccine was instilled into each nostril. After randomization, all dogs except dog 10, underwent 1 week of desensitization training to allow for low-stress vaccination.

#### Study 2: Between-Subjects Intranasal Vaccine Study

Each dog was vaccinated based on the days they would be tested on the scent wheel. The M/Th running group received their treatment on a Monday. The T/F group received their treatment on the next day (Tuesday). The treatments were given in the same order as the dogs in each group completed training and testing on the wheel. Each dog was individually brought into a room with a veterinarian. The control dogs did not receive any treatment. They were allowed to roam around the room and the veterinarian then called for the dog to be taken back to their kennel. The diluent treatment group received saline intranasally. The vaccine treatment group received the intranasal *Bordetella bronchiseptica* vaccine. The diluent and vaccine group dogs were each scored from 0 to 3 by the veterinarian based on the amount of fluid that splashed outside the dog's nose. If all the liquid from the treatment ended up in the dog's nose, they were scored a 0. If very little liquid ended up entering the dog's nose, they were scored a 3. This was a non-normally distributed ordinal variable, where most dogs scored a 1 or 2.

### Post-vaccination Testing

#### Study 1: Crossover Oral and Intranasal Vaccine Study

Following baseline and vaccination, sequential testing on the scent wheel was utilized to determine olfactory detection acuity, and the lowest dissipation rate detected for each dog on any given test day was recorded. The dogs were initially tested on UDC inside of a 4MIL LDPE bag housed in a 3 x 5 inch bag containing a 0.02 cm^2^ hole that was in a 4 x 6 inch bag containing a 0.004 cm^2^ hole. If the dog was unable to detect the odor during the trial (if the dog had three false positive responses or conducted 40 s of active searching without a behavior change in UDC odor), the dog was tested sequentially with samples with larger permeation pores on subsequent trials until the dog was successfully able to detect the odor and elicit a positive response (next level of UDC was a 4MIL LDPE bag housed in a 3 x 5 inch bag containing a 0.08 cm^2^ hole in a 4x6 inch bag containing a 0.004 cm^2^ hole). Odor detection acuity was determined after a positive response to the lowest concentration of UDC. A degradation in olfactory detection ability was determined by a decrease in the detection acuity on any given day as compared to the baseline olfactory detection acuity.

#### Study 2: Between-Subjects Intranasal Vaccine Study

Testing occurred on days 0, 7, 10, 11, 14, 21, 24, 28, and 29 post-vaccination. Some of these intervals were shifted from the original study design due to scheduling conflicts or inclement weather conditions. The target odor (the lowest concentration of UDC) was randomly placed in either port 3, 5, or 7, which were the positions utilized during training and baseline assessments. Position of target odor varied between testing days but not between dogs. If dogs passed by the target odor without exhibiting their trained alert, the target odor was replaced with a higher concentration of UDC (1/16 x needle) in the same position. The dogs were then sent back in once more, and the session was ended after the second trial, regardless of whether or not the dog exhibited their trained alert upon smelling the target odor.

### Data Collection

In addition to video recorded data, live observational data was recorded while dogs were searching for odor in the case of both studies. The dogs' response to odor, trial duration, and behavioral observations were recorded along with location of samples, start and end time of each trial, the permeation pore size of the UDC, and additional factors relevant to individual dogs (i.e., current medications, medical conditions, and environmental factors).

### Behavior Analysis

While different programs were used in each study to carry out behavior coding, the processes were similar. All coded parameters and behaviors are defined in Supplementary Table 5. Due to variations in canine behavior during searches, a behavior change indicating the dog was responding to the odor could have occurred before a dog provided its trained final response to the odor, so these occurrences were coded separately.

Behaviors of the dogs during each trial were scored using a motivation ethogram developed to quantify each individual dog's motivation to perform the assigned task. Positive motivation factors (PMFs) were defined as behaviors that correlated to a high motivational drive for the assigned task, consisting of over-stimulation or over-excitement displacement behaviors. Conversely, negative motivation factors (NMFs) were defined as behaviors that correlated to a low motivational drive, which consisted of disinterest or distraction displacement behaviors (23). In Study 1, video analysis was performed with Noldus Observer XT 14. Three trials were not coded via video analysis due to missing videos- Joey, Baseline Intranasal, Joey, 4 h Post-Oral, and Ellie, 4 h Post-Oral. The first two were coded using raw data, while the latter is a missing data point due to removal of dog before testing could be completed. In Study 2, observations were coded using BORIS by a research assistant who was blind to treatment groups coded each dog's session, which was used to examine the effect of positive and negative motivating factors on duration of time to odor (24). Reliability coding took place in both studies. We examined whether the vaccine affected dogs' search duration over the testing period. Search duration in this analysis was defined as the total duration of time from the dog entering the wheel room at the start of the trial to the dog exiting the wheel room after alerting on the positive UDC sample. Trials where the dog failed to alert on the positive sample were excluded, as failing to alert meant the dog checked all the ports after the positive port and only then exited the wheel, and as such these trials were a different length. We also analyzed the dogs for potential behavioral differences during their wheel search as a result of the vaccine.

## Statistics

Statistical analyses were carried out in R version 4.0.0.

### Study 1: Crossover Oral and Intranasal Vaccine Study

#### Model 1: How Is Search Duration Affected by Vaccination Status and Test Day?

Using the R package lme4, a linear mixed-effect model was run for duration to behavior change with treatment type (oral vs. intranasal), experimental session (session 1 vs. session 2), and timepoint as fixed effects with a three-way interaction, and dog as a random effect (25). This model was used to investigate the potential effects of treatment type, session, and timepoint on the duration to behavior change on the target odor.

#### Model 2: What Is the Effect of Behavioral Motivators on Search Duration?

To examine the effect of the presence of positive and negative behavioral motivators on duration to behavior change, a second linear-mixed effect model was run with the positive (Present or Absent) and negative (Present or Absent) motivators as fixed effects with a two-way interaction, and dog as a random intercept.

### Study 2: Between-Subjects Intranasal Vaccine Study

#### Model 1: Baseline Data vs. Test Data

A binomial mixed-effects regression was used to examine whether the treatment group of the dogs and whether testing occurred at baseline or during test phase affected whether dogs correctly alerted on the lowest concentration of UDC, or whether they had to switch to a higher concentration of UDC (see Supplementary Table 3). This was done using the lmer() function in R (25). Vaccination status and baseline vs. test served as fixed effects, and the dog was a random effect.

#### Model 2: How Does Vaccination Status and Test Date Impact Dogs' Failure to Alert on the Lowest Concentration of UDC?

A binomial mixed-effects regression was used to examine whether the treatment group of the dogs and the timepoint tested influenced dogs' alert on the lowest concentration of UDC (see Supplementary Table 3). This was done using the lmer() function in R (25). Vaccination status and timepoint served as fixed effects, and the dog was a random effect.

#### Model 3: How Is Search Duration Affected by Vaccination Status and Test Day?

A linear mixed-effect model of search duration was used for this analysis, created with the lme() function in R (25). Predictor variables included between-subjects factor Vaccine Status (Vaccinated, Diluent, or Control, with Vaccinated as the baseline) and Target Odor Location (3, 5, or 7) and within-subjects continuous factor Day of Test (Baseline Day 1 (coded as −14), Baseline Day 2 (coded as −12), Baseline Day 3 (coded as −7), 0, 6, 7, 10, 11, 14, 21, 24, 28, or 29).

#### Model 4: What Is the Effect of Behavioral Motivators on Search Duration?

To examine the effect of the presence of positive and negative motivating behaviors on duration to behavior change, a linear-mixed effect model was run with the positive (Present or Absent) and negative (Present or Absent) motivating behaviors as fixed effects with a two-way interaction, and dog as a random intercept.

## Results

The results of the initial cross-over study and the between-subjects study will be discussed separately.

### Study 1: Crossover Oral and Intranasal Vaccine Study

#### Model 1: How Is Search Duration Affected by Vaccination Status and Test Day?

All dogs detected UDC with the lowest dissipation rate, except for one dog each on 1 day post (Dog 13), 7 days post (Dog 5), and 10 days post (Dog 7) in the intranasal treatment group and one dog on day 7 (Dog 12) and one dog on both days 1 and 7 (Dog 1) in the oral treatment group, which recognized the next highest dissipation rate. As such, trials conducted with the higher dissipation rate UDC were not included in this statistical model since there were so few.

A linear mixed-effect model was run for duration to behavior change with treatment (oral vs. intranasal), experimental session (session 1 vs. session 2), and timepoint as fixed effects with a three-way interaction, dog as a random intercept, and a random slope of experimental session on dog.

A likelihood ratio test compared the model with and without the random slope. The test suggests that there is no significant difference between these models X^2^(1) = 1.34, p = 0.719. AIC scores suggest the model without the slope is a better fit (AIC of 1498 vs. AIC of 1502); as such, we removed the random slope from the model.

There was a main effect of Treatment, ß_1_ = 59.48, t(162) = 4.11, *p* = 0.0001, such that dogs who received the oral vaccine had a longer duration to behavior change overall than dogs who received the intranasal vaccine (oral vaccine: 20.23 sec; intranasal vaccine: 19.87 sec). There was no main effect of experimental session, ß_1_ = 8.75, t(162) = 1.39, *p* = 0.167, or timepoint, ß_1_ = −0.8, t(162) = −0.34, *p* = 0.733.

The three-way interaction between treatment (oral vs. intranasal), experimental session (session 1 vs. session 2), and timepoint (all points vs. baseline) was not significant, ß_1_ = 2.21, t(162) = 1.07, p = 0.287. The interaction between treatment and session was not significant, ß_1_ = −6.02, t(162) = −1.83, p = 0.069.

**Figure 2:**
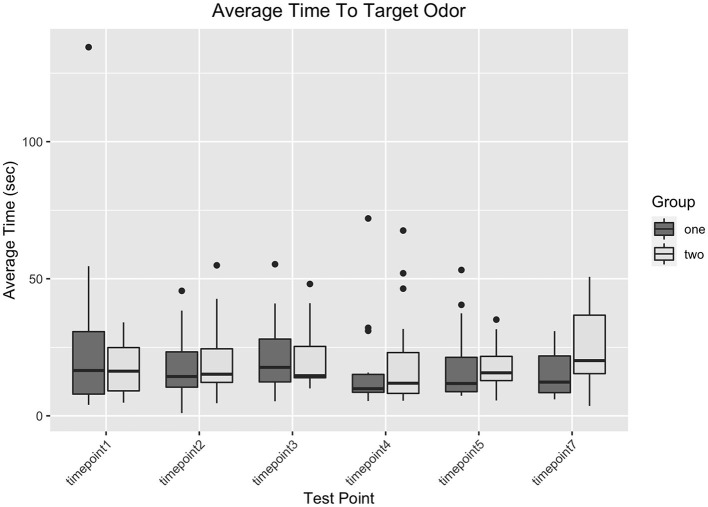
A graph of dogs' search duration in seconds at each timepoint in the study grouped by the current treatment they were receiving.

However, there was a significant interaction between experimental session and treatment, ß_1_ = −32.87, t(162) = −3.55, p = 0.0005, where in experimental session 1, dogs that received the oral vaccination had a longer duration to behavior change (overall, not by timepoint), while in experimental session 2, dogs that received the intranasal vaccination had a longer duration to behavior change (overall, not by timepoint). The group of dogs that were assigned the treatment order of oral then intranasal were generally slower to find the target odor than the dogs that were assigned the treatment order of intranasal then oral. Figure 2 shows a graph of search duration by vaccination status and test day.

#### Model 2: What Is the Effect of Behavioral Motivators on Search Duration?

Forty-eight videos (25%) were randomly selected from a total of 192 and were re-analyzed by a second observer familiar with animal behavior and scent detection dogs to calculate the reliability of the coding scheme. There was a reliability frequency for point events of 78.0% and a reliability frequency of duration events of 83.5%. The reliability frequency for the number of total canine behavior changes in a trial (including both true positives and false positives) was 82.4%.

A second model was used to examine the effect of positive and negative behavioral motivation on duration to behavior change on odor. Table 1 shows the distribution of trials in which dogs displayed positive and NMFs. Individual positive and negative motivating behaviors did not occur frequently enough for an individual analysis of their effect, and the distribution of the number of positive and negative motivating behaviors per trial was non-normal. As such, we instead coded the data into two categorical variables: the presence or absence of PMFs, and the presence or absence of NMFs.

**Table 1:** Count of trials with and without positive and negative behavioral motivators.

	**PMF absent**	**PMF present**
NMF Absent	102	8
NMF Present	60	15

There was a significant main effect of the presence of positive motivating factors, ß_1_ = 13.94, t(166) = 2.49, p = 0.014, such that dogs' duration to behavior change was longer if a positive motivating behavior occurred in the trial. There was a significant main effect of the presence of negative motivating factors, ß_1_ = 8, t(166) = 2.25, p = 0.0005, such that dogs' duration to behavior change was longer if a negative motivating behavior occurred in the trial. There was no interaction, ß_1_ = 1.19, t(166) = 0.183, *p* = 0.855.

### Study 2: Between-Subjects Intranasal Vaccine Study

This study examined the effect of the intranasal Bordetella vaccine on dogs' performance in odor detection in the 28 days after vaccine administration. This study used a between-groups design, as the cross-over design in the prior study showed that administration of two vaccines within a month period may have affected the dogs' search time. While Splash factor (an estimated amount of the vaccine that did not enter the dogs' nose due to “splashing” out when the dog moved) was measured, a Mann-Whitney U test shows that there is no difference between the vaccine group's splash factor score (median = 1) and the diluent group's splash factor score (median = 1), W=281,130, *p* = 0.48. As a result, splash factor scores were not included in any analysis.

#### Model 1: Baseline Data vs. Test Data

During test trials, the vaccinated group (nine dogs) required a switch to a higher concentration 22 times out of 63 test trials (34%). The diluent group (10 dogs) required a switch to a higher concentration 19 times out of 70 test trials (27%). The control group (5 dogs) required a switch to 11 times out of 35 test trials (31%). In the baseline trials, in which the vaccinated group required a switch to the higher concentration 17 times out of 81 trials (21%), The diluent group required a switch to a higher concentration 13 times out of 90 test trials (14%) The control group required a switch to 6 times out of 45 test trials (13%). There is no significant difference between the baseline and test days in terms of higher concentration switches (X^2^ = 0.26, z value = 0.37, p = 0.71). There is no effect of vaccination groups (X^2^ = 0.36, z value = 0.73, p = 0.61) and no interaction between the variables ((X^2^ = 0.21, z value = 0.23, p = 0.81).

#### Model 2: How Does Vaccination Status and Test Date Impact Dogs' Failure to Alert on the Lowest Concentration of UDC?

There was no main effect of Timepoint, X^2^ = 0.04, z value = 0.969, p = 0.33. There was no main effect of Vaccination Status (Diluent vs. Control: X^2^ = −0.1, z value = −0.08, p = 0.93; Vaccine vs. Control: X^2^ = 0.93, z value = 0.73, p = 0.46; Vaccine vs. Diluent: X^2^ = 1.03, z value = 0.99, p = 0.32).

There was no interaction between Time and Vaccination Status (Time x Vaccine vs. Diluent: X^2^ = −0.03, z value = −0.74, p = 0.46; Time x Control vs. Diluent, X^2^ = 0.001, z value = 0.01, p = 0.99; Time x Vaccine vs. Control, X^2^ = −0.03, z value = −0.63, p = 0.53).

#### Model 3: Search Duration for Vaccinated vs. Diluent and Control Groups by Testing Day

There was no main effect of Vaccine Status: Diluent; there was no significant difference between dogs who received the diluent and dogs who received the vaccine, ß_1_ = 1.752, t(21) = 0.871, p = 0.394. There was no main effect of Vaccine Status: Control; there is no significant difference between control dogs and dogs who received the vaccine, ß_1_ = 1.896, t(21) = 0.813, p = 0.426.

There was no main effect of Day; the dogs' search duration did not differ based on the day of testing, ß_1_ = 0.513, t(292) = 0.531 p = 0.596. There was a main effect of Target Odor Location, ß_1_ = 0.746, t(292) = 3.154, p = 0.002, such that search duration was longer as the positive port number increased; this was an intuitive finding, as it would naturally take longer to arrive at a positive sample located in port 7 of the wheel than a positive sample in port 3.

**Figure 3:**
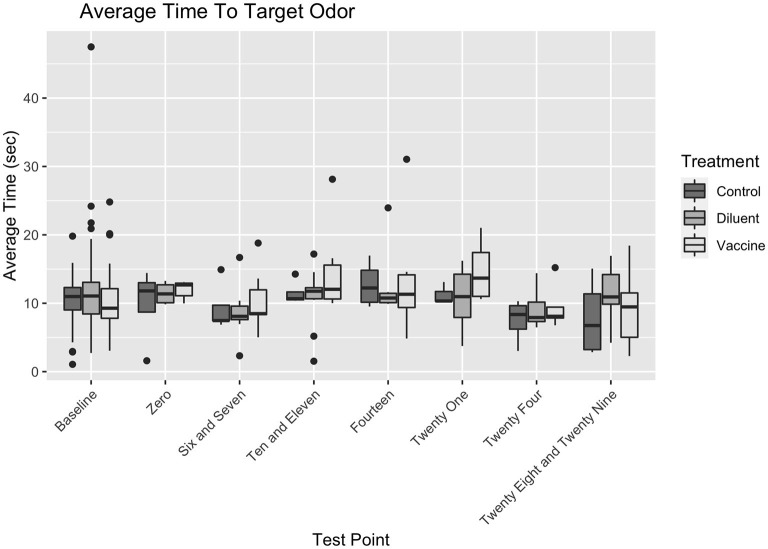
Duration at port by vaccination status and testing day.

There were no significant interactions between any variables. Vaccine Status did not affect search duration differently based on the Day of Test (for Day x Control vs. Vaccine, ß_1_ = −0.15, t(292) = −1.001, p = 0.318; for Day x Diluent vs. Vaccine, ß_1_ = −0.126, t(292) = −0.955, p = 0.34). Vaccine Status also did not affect search duration differently based on Positive Port Location (for Positive Port Location x Control vs. Vaccine, ß_1_ = −0.478, t(292) = −1.283, p = 0.2; for Day x Diluent vs. Vaccine, ß_1_ = −0.289, t(292) = −0.902, p = 0.358). The Positive Port Location did not affect search duration differently on different Day of Tests, ß_1_ = −0.003, t(292) = −0.181, p = 0.856. There were no significant three–way interactions between Vaccine Status, Day of Test, and Target Odor Location (for Day x Target x Control vs. Vaccine, ß_1_ = 0.146, t(292) = 0.554, p = 0.58; for Day x Target x Diluent vs. Vaccine, ß_1_ = 0.007, t(292) = 0.326, p = 0.745). Figure 3 shows the search duration as function of treatment group and testing day.

#### Model 4: What Is the Effect of Behavioral Motivators on Search Duration?

Table 2 shows the distribution of trials in which dogs displayed PMFs and NMFs. As in the first experiment, individual positive and negative motivating behaviors did not occur frequently enough for an analysis of their effect, and the distribution of the number of positive and negative motivating behaviors per trial was non-normal. As such, we instead coded the data into two categorical variables: the presence or absence of PMFs, and the presence or absence of NMFs.

**Table 2:** Count of trials with and without positive and negative behavioral motivators.

	**PMF absent**	**PMF present**
NMF Absent	196	127
NMF Present	82	61

There was no main effect of the presence of PMFs, ß_1_ = 0.29, t(440) = 0.33, p = 0.74. There was a significant main effect of the presence of NMFs, ß_1_ = 3.59, t(440) = 3.8, p = 0.0002, such that dogs' duration to behavior change was longer if a negative motivating behavior occurred in the trial. There was no interaction, ß_1_ = −0.87, t(440) = −0.6, p = 0.547. This differs from the result in Study 1, where we found an effect of both PMFs and NMFs.

## Discussion

Canine olfactory detection requires odor delivery, processing and recognition including display of the trained response. Odor delivery can be influenced by the dog's behavior and motivation to seek out the source. Once in the proximity of the odor source, the odorant molecules must enter and transit the nasal passages and bind to olfactory receptors in the olfactory epithelium. Interference with odor molecule transit to the receptors has been referred to as conductive anosmia/hyposmia and can result from local inflammation or obstruction to airflow. After binding to the receptors, the olfactory nerves deliver the signal to the olfactory bulb and the information is relayed to the cortex to trigger a trained response. Any interference with nerve conduction, whether from the peripheral olfactory nerves or the central processing could potentially impair olfaction (6, 26). The demonstration of the trained response to an odor depends on the cognitive processing and the expression of the trained behavior (27). Neurologic, metabolic or behavioral factors impacting cognition and behavior could impair this final stage of odor detection.

In the initial cross-over study of 16 detection dogs, we were unable to detect a systematic effect of vaccination on the ability to detect the lowest concentration of odor but did identify factors that influenced the time it took to identify the odor, i.e., search efficiency. Despite randomization, the groups performed differently; the group given the oral vaccine in session 1 followed by the intranasal vaccine in session 2 took longer to find the target odor than their counterparts that received the intranasal vaccine in session 1 and the oral vaccine in session 2. One potential explanation could be conductive hyposmia due to a compounding effect of immune stimulation resulting from the short (28 day) washout period between vaccine administrations in session 2, with the order of vaccination method playing a role. There was also a significant effect of positive and negative motivating factors in regards to duration to behavior change. However, relatively few PMFs were noted in the trials, given the larger sample size and larger number of PMFs in Study 2, the effect was no longer present.

Given the effect seen from administering the vaccine twice within a month, a second between-subjects placebo-controlled study was conducted to examine the effect of a single dose of intranasal vaccine on olfaction and search behavior over a 29-day period. The intranasal vaccine was specifically used due to its broad use and higher potential for the intranasal vaccine to lead to decreased olfactory capabilities. In this study, there was no evidence to support intranasal *Bordetella bronchiseptica* vaccination impacted dogs' olfactory sensitivity or efficiency (time to target odor). Counts of which dogs required higher concentration for correct detection and their vaccination group can be found in Supplementary Table 5.

Because all dogs were thoroughly trained on this target odor prior to the studies, and baselines for each dog were available for comparison, it is unlikely that difficulty detecting or inability to detect the lowest concentration of UDC is a result of impaired olfactory ability. This suggests that the vaccination itself had no measurable impact on the dogs' abilities to correctly distinguish and detect a target odor in either study for up to 28 days post vaccination, and that prolonged search duration was primarily a result of dogs exhibiting behavioral indicators of discomfort, boredom, or stress within the environment (see Figure 3).

We examined how behavioral parameters could affect search efficiency either by impacting the behavior required to access to the odor or to demonstrate the trained response. To account for the varied individualistic search behaviors and behavioral responses of each dog and their influence on behavior change in odor, observed behaviors were qualified, quantified, and categorized as of “positive motivation” or “negative motivation”. The negative factors were particularly chosen to represent a decreased motivation to complete the detection task, as they are indicative of distraction, stress, and frustration and are known to affect the success of trained scent detection dogs. Positive factors consisted of stress and redirection behaviors that may not have affected their search time but may have increased their anxiety and arousal, which also has a noteworthy impact on the ability of a scent detection dog to find their target odor, especially if a dog already has a high level of arousal before they are asked to search for odor (23). Overall, both studies found that higher negative motivation was significantly correlated with a longer duration to behavior change on odor, whereas there were mixed results on the effect of positive motivation. The first study found that positive motivation was correlated with a longer duration to behavior change on odor, while in the second study, only the presence of NMFs significantly increased the duration of time to behavior change. Given the low number of PMF occurrences in the first study, it is likely that the second study represents a more accurate result.

Based on the observed effects of positive motivation and negative motivation in this study, future olfactory detection studies involving detection dogs should also account for search behavior in addition to olfactory capabilities due to the observed effect of behavior on the dogs' search efficiency. Future studies should also narrow down potential behaviors that could lead to changes in search behavior; while the broader ethogram we used identified that behavior plays a role, it is still unclear which behaviors specifically correlate with dogs' detection abilities and search efficiency.

A potential limitation of this study was the use of UDC as the target odor. In an operational setting, detection dogs may be searching for a more complex or variable odor than UDC (e.g., explosives, live humans, human remains, accelerants). UDC is a training odor that is utilized to teach dogs and puppies detection skills. The concentration of UDC while the lowest concentration available, is still an apparently easy scent for dogs to detect. As such, it is possible that dogs could be able to detect and alert on the test UDC even if their olfaction was affected. However, in the second study, dogs in all treatment groups did occasionally fail to alert on the test UDC; the test target odor was not alerted on and thus switched to the higher (1/16 x needle) concentration 53 times out of the total 168 trials completed (31.5%). The dogs did not fail to alert in any systematic pattern; rather, it was randomly distributed, and some dogs were more prone to fail to alert than others. Since the dogs still occasionally struggled to correctly alert on the test UDC, it suggests that the UDC concentration was not a contributing factor to the null outcome of this study.

Another limitation in both studies is the lack of data collected on nasal tract inflammation. One method being currently utilized is the measurement of concentration of odorant binding proteins (OBP) utilizing leukosorb paper in direct contact to the olfactory epithelium in humans (28). This method, as proven legitimate by Yoo et al., might be utilized to measure inflammation of the sinonasal tract in all participants before and after vaccination. This would provide a quantifiable component to support the conclusion that the inflammation resulting from intranasal vaccination is not enough to prevent a trained detection dog from performing olfactory based tasks effectively.

Inclement weather conditions resulted in a closure of the University and a shift in some of the intervals on which dogs were tested, which provides another source of potential error for the second study. Having a shift in schedule may have affected dogs' behavior. However, these shifts never resulted in moving a test day more than one day off schedule; as such, the weather likely did not create any appreciable difference in how vaccination affected the dogs' olfactory abilities.

In summary, odor threshold was not influenced by any vaccine strategy, a single intranasal or oral Bordetella vaccine did not impact detection efficiency, but an intranasal vaccine at 28 days following an oral vaccine did result in a small but statistically significant increase in time to detection. Clinical recommendations would be to administer the Bordetella vaccine according to the recommended 6–12 month frequency. As with all medications in detection dogs, handlers and veterinarians should be alert to individual performance changes following treatment, but based on these studies, the risk of hyposmia with Bordetella vaccine is low. Behavior of the dog also influences the search efficiency and should be considered in studies and clinical scenarios evaluating olfactory function.

## Data Availability Statement

The raw data supporting the conclusions of this article will be made available by the authors, without undue reservation.

## Ethics Statement

The animal study was reviewed and approved by University of Pennsylvania Institutional Animal Care and Use Committee. Written informed consent was obtained from the owners for the participation of their animals in this study.

## Author Contributions

RB, JE, PK, RF, and JG assisted in the implementation of the first study. JE and CO designed the study with assistance from PK. RB wrote an initial version of the introduction, methods, results, and discussion. AM designed the statistical models, wrote the final draft of the results, designed the statistical models, and wrote the results. AC wrote the final draft of the introduction, methods, and discussion. RF and JG assisted in coding videos. AM, JE, and CO designed the second study. AC and SK assisted in the implementation of the second study. AC and AM wrote the methods and final discussion. All authors contributed to the article and approved the submitted version.

## Funding

This study was fully funded by Zoetis.

## Conflict of Interest

The authors declare that the research was conducted in the absence of any commercial or financial relationships that could be construed as a potential conflict of interest.

## Publisher's Note

All claims expressed in this article are solely those of the authors and do not necessarily represent those of their affiliated organizations, or those of the publisher, the editors and the reviewers. Any product that may be evaluated in this article, or claim that may be made by its manufacturer, is not guaranteed or endorsed by the publisher.
