# Supplementary material for: Effects of Intranasal and Oral Bordetella bronchiseptica Vaccination on the Behavioral and Olfactory Capabilities of Detection Dogs
Source: Front Vet Sci. 2022 May 18;9:882424. doi: 10.3389/fvets.2022.882424 (PMC9159271; doi:10.3389/fvets.2022.882424)
Supplement: Supplementary file 1 [file Table_1.docx]

**Supplementary Table 1**

*Dogs Utilized in Study 1*

| **Dog** | **Breed** | **Sex** | **Age at Start of Study (in years)** | **First Treatment** |
| --- | --- | --- | --- | --- |
| Murphy | Dutch Shepherd | M | 1.02 | Intranasal |
| Willow | Labrador Retriever | F | 0.89 | Intranasal |
| Déjà vu | German Shepherd | F | 1.45 | Intranasal |
| Lucy | Dutch Shepherd | F | 1.02 | Intranasal |
| Jolie | Dutch Shepherd | F | 1.02 | Intranasal |
| Joey | Dutch Shepherd | M | 1.02 | Intranasal |
| Jenner | Labrador Retriever | M | 0.72 | Intranasal |
| Ivey | German Shepherd | F | 1.41 | Intranasal |
| Odysseus | Dutch Shepherd | M | 1.02 | Oral |
| Hawk* | Dutch Shepherd | M | 1.11 | Oral |
| Tanner | Labrador Retriever | M | 0.71 | Oral |
| Tallon | Labrador Retriever | M | 0.71 | Oral |
| Blitz | Belgian Malinois | M | 0.65 | Oral |
| Cody | German Shepherd | M | 1.12 | Oral |
| Ellie | German Shepherd | F | 1.19 | Oral |
| Callie | Dutch Shepherd | F | 1.02 | Oral |

*Run by Familiar Handler
