# Supplementary material for: Effects of Intranasal and Oral Bordetella bronchiseptica Vaccination on the Behavioral and Olfactory Capabilities of Detection Dogs
Source: Front Vet Sci. 2022 May 18;9:882424. doi: 10.3389/fvets.2022.882424 (PMC9159271; doi:10.3389/fvets.2022.882424)
Supplement: Supplementary file 2 [file Table_2.docx]

Supplementary Table 2

*Dogs Utilized in Study 2*

| **Dog** | **Breed** | **Sex** | **Age at Start of Study (in years)** | **Treatment Group** |
| --- | --- | --- | --- | --- |
| Bacco | Belgian Malinois | M | 0.58 | Vaccine |
| Bo | Doberman | M | 2.00 | Vaccine |
| Cody* | German Shepherd | M | 3.75 | Vaccine |
| Fury | Belgian Malinois | M | 1.33 | Vaccine |
| Griz* | German Shepherd | M | 5.50 | Vaccine |
| Ivey | German Shepherd | F | 2.67 | Vaccine |
| Pacy* | Labrador Retriever | F | 7.00 | Vaccine |
| Roxie* | Labrador Retriever | F | 6.00 | Vaccine |
| Ugo | Labrador Retriever | M | 1.25 | Vaccine |
| Bailey | Labrador Retriever | M | 0.83 | Diluent |
| Bobbie | German Shepherd | F | 4.92 | Diluent |
| Charlie* | Labrador Retriever | M | 4.00 | Diluent |
| Coyote | Belgian Malinois | M | 0.75 | Diluent |
| Lucy | Dutch Shepherd | F | 3.67 | Diluent |
| Osa | German Shepherd | F | 6.75 | Diluent |
| Sky | Dutch Shepherd | F | 0.92 | Diluent |
| Sheridan | German Shepherd | M | 1.08 | Diluent |
| Uzza | Labrador Retriever | F | 1.25 | Diluent |
| Tuukka* | Border Collie Mix | F | 7.00 | Diluent |
| Crunch* | Pembroke Welsh Corgi | M | 4.00 | Control |
| Gunner | Dutch Shepherd | M | 0.92 | Control |
| Helen* | Labrador Retriever | F | 3.50 | Control |
| Rico* | German Shepherd | M | 6.00 | Control |
| Toby | Small Munsterlander | M | 2.50 | Control |

*Run by Familiar Handler
