# Supplementary material for: Effects of Intranasal and Oral Bordetella bronchiseptica Vaccination on the Behavioral and Olfactory Capabilities of Detection Dogs
Source: Front Vet Sci. 2022 May 18;9:882424. doi: 10.3389/fvets.2022.882424 (PMC9159271; doi:10.3389/fvets.2022.882424)
Supplement: Supplementary file 3 [file Table_3.docx]

Supplementary Table 3

*Baseline detection scores and mechanics scores for dogs in the second study*

This table shows the dogs’ baseline accuracy on odor (d-prime detection score) and an assessment of their mechanics on the scent wheel prior to beginning the second study.

| Dog | D-prime detection score | Wheel mechanics score |
| --- | --- | --- |
| Bacco | 3.09 | 18.00 |
| Bailey | 3.71 | 14.67 |
| Bo | 3.94 | 20.33 |
| Bobbie | 4.04 | 7.00 |
| Charlie | 4.65 | 10.50 |
| Cody | 3.74 | 11.00 |
| Coyote | 3.79 | 17.33 |
| Crunch | 3.64 | 15.50 |
| Fury | 3.58 | 11.00 |
| Griz | 4.39 | 5.50 |
| Gunner | 3.61 | 15.33 |
| Helen | 4.83 | 14.00 |
| Ivey | 5.07 | 2.67 |
| Lucy | 4.17 | 3.33 |
| Osa | 4.51 | 2.00 |
| Pacy | 3.45 | 6.33 |
| Rico | 3.77 | 6.00 |
| Roxie | 3.59 | 13.50 |
| Sheridan | 3.63 | 18.67 |
| Sky | 3.42 | 17.00 |
| Toby | 4.03 | 1.33 |
| Tuukka | 3.51 | 9.00 |
| Ugo | 3.68 | 8.67 |
| Uzza | 3.13 | 6.33 |
