# Supplementary material for: Effects of Intranasal and Oral Bordetella bronchiseptica Vaccination on the Behavioral and Olfactory Capabilities of Detection Dogs
Source: Front Vet Sci. 2022 May 18;9:882424. doi: 10.3389/fvets.2022.882424 (PMC9159271; doi:10.3389/fvets.2022.882424)
Supplement: Supplementary file 4 [file Table_4.docx]

Supplementary Table 4

*Observer XT Data Analysis Definitions*

This table shows and defines the different behaviors on our study videos that were coded in BORIS, and what category of behavior they were considered (General behavior, negative motivating factor, or positive motivating factor).

| Behavior Type | Behavior | Point / Duration Event | Definition |
| --- | --- | --- | --- |
| General | Behavior change (UDC odor) | Point | behavior change (stop, stare, turn around, hesitate, prolonged port interaction) at port containing UDC odor |
| General | Behavior change (false) | Point | behavior change (stop, stare, turn around, hesitate, prolonged port interaction) at port containing UDC control or distractor |
| General | Pause in trial | Point | removal of dog from trial room due to reset, while resolving a trial issue, or in between trials |
| General | Trial duration | Duration | time period (s) from dog entering trial room to dog giving final alert, subtracting any pauses in trial |
| General | Trial duration to behavior change (odor) | Duration | time period (s) from dog entering trial room to behavior change at odor, subtracting any pauses in trial |
| Positive Motivating Factor | Vocalization | Point | vocalizes during trial |
| Positive Motivating Factor | Jump on wheel | Point | dog jumps on top of the search wheel |
| Positive Motivating Factor | Paw at port | Point | dog paws at any part of the wheel |
| Positive Motivating Factor | Running | Duration | dog runs/jogs during active search, which may lead to skidding, slipping, and/or hitting the wheel while searching |
| Positive Motivating Factor | Reset | Point | dog is recalled by trainer during search due to behavior, given a brief pause, re-issued ‘search’ command from trainer, and sent back into the room to continue trial search |
| Negative Motivating Factor | Stare at observer | Point | dog glances where trainer/handler is located |
| Negative Motivating Factor | Exit attempt | Point | dog attempts to exit barriers or exit trial room door during trial |
| Negative Motivating Factor | Interaction with object | Point | dog interacts with object other than the wheel (floor, barrier) during trial |
| Negative Motivating Factor | Pause in active search (distraction) | Duration | dog ceases active search for odor and is not interacting with the wheel or its ports or attempting to alert |
| Negative Motivating Factor | Resend | Point | dog attempts to exit trial area through main door, is re-issued ‘search’ command from trainer, and is sent back into the room to continue trial search |
