# Supplementary material for: Effects of Intranasal and Oral Bordetella bronchiseptica Vaccination on the Behavioral and Olfactory Capabilities of Detection Dogs
Source: Front Vet Sci. 2022 May 18;9:882424. doi: 10.3389/fvets.2022.882424 (PMC9159271; doi:10.3389/fvets.2022.882424)
Supplement: Supplementary file 5 [file Table_5.docx]

Table 5

*Dogs’ treatment groups and times they required higher concentration of UDC*

This table shows the number of times that dogs failed to alert on the lowest concentration of UDC and the timepoints at which they failed.

| Dog | Treatment group | # of times higher concentration required (because dog failed initial trial) | Timepoints higher concentration required (because dog failed initial trial) |
| --- | --- | --- | --- |
| Bacco | Vaccine | 0 |  |
| Bo | Vaccine | 4 | T0, T10, T21, T24 |
| Cody | Vaccine | 3 | T10, T21, T28 |
| Fury | Vaccine | 1 | T21 |
| Griz | Vaccine | 5 | T0, T14, T21, T24, T28 |
| Ivey | Vaccine | 0 |  |
| Pacy | Vaccine | 5 | T0, T14, T21, T24, T28 |
| Roxie | Vaccine | 3 | T7, T21, T24 |
| Ugo | Vaccine | 1 | T21 |
| Bailey | Diluent | 3 | T0, T7, T24 |
| Bobbie | Diluent | 1 | T28 |
| Charlie | Diluent | 6 | T0, T7, T10, T21, T24, T28 |
| Coyote | Diluent | 1 | T24 |
| Lucy | Diluent | 1 | T0 |
| Osa | Diluent | 3 | T7, T14, T28 |
| Sky | Diluent | 0 |  |
| Sheridan | Diluent | 2 | T21, T24 |
| Uzza | Diluent | 0 |  |
| Tuukka | Diluent | 2 | T24, T28 |
| Crunch | Control | 2 | T14, T21 |
| Helen | Control | 2 | T21, T28 |
| Gunner | Control | 0 |  |
| Rico | Control | 5 | T7, T10, T21, T24 |
| Toby | Control | 2 | T14, T21 |
